# Integration of egocentric and allocentric information during memory-guided reaching to images of a natural environment

**DOI:** 10.3389/fnhum.2014.00636

**Published:** 2014-08-25

**Authors:** Katja Fiehler, Christian Wolf, Mathias Klinghammer, Gunnar Blohm

**Affiliations:** ^1^Department of Experimental Psychology, Justus-Liebig-UniversityGiessen, Germany; ^2^Canadian Action and Perception Network (CAPnet), Centre for Neuroscience Studies, Queen’s UniversityKingston, ON, Canada

**Keywords:** reference frame, reaching, natural scene, allocentric information, egocentric information, human

## Abstract

When interacting with our environment we generally make use of egocentric and allocentric object information by coding object positions relative to the observer or relative to the environment, respectively. Bayesian theories suggest that the brain integrates both sources of information optimally for perception and action. However, experimental evidence for egocentric and allocentric integration is sparse and has only been studied using abstract stimuli lacking ecological relevance. Here, we investigated the use of egocentric and allocentric information during memory-guided reaching to images of naturalistic scenes. Participants encoded a breakfast scene containing six objects on a table (local objects) and three objects in the environment (global objects). After a 2 s delay, a visual test scene reappeared for 1 s in which 1 local object was missing (= target) and of the remaining, 1, 3 or 5 local objects or one of the global objects were shifted to the left or to the right. The offset of the test scene prompted participants to reach to the target as precisely as possible. Only local objects served as potential reach targets and thus were task-relevant. When shifting objects we predicted accurate reaching if participants only used egocentric coding of object position and systematic shifts of reach endpoints if allocentric information were used for movement planning. We found that reaching movements were largely affected by allocentric shifts showing an increase in endpoint errors in the direction of object shifts with the number of local objects shifted. No effect occurred when one local or one global object was shifted. Our findings suggest that allocentric cues are indeed used by the brain for memory-guided reaching towards targets in naturalistic visual scenes. Moreover, the integration of egocentric and allocentric object information seems to depend on the extent of changes in the scene.

## Introduction

When reaching to a visual target in a naturalistic environment, the brain can make use of absolute or relative spatial information for reach planning. This can be formalized in terms of two broad classes of reference frames: an egocentric reference frame that represents the absolute position of an object with respect to the observer and an allocentric reference frame coding the position of an object relative to other objects in the environment (Colby, 1998). While egocentric reference frames depend on eye, head, body, etc. position and orientation, allocentric reference frames are relatively observer-invariant. It is well known that for goal-directed reaching movements, a gaze-dependent, egocentric reference frame is used preferentially as demonstrated by electrophysiological studies in monkeys (Batista et al., 1999; Buneo et al., 2002) and behavioral (Henriques et al., 1998; Medendorp and Crawford, 2002; Fiehler et al., 2011) and brain imaging studies (Medendorp et al., 2003; Bernier and Grafton, 2010) in humans.

Despite the dominance of gaze-dependent representations for reach planning, allocentric information also contributes to the encoding of reach target location. For example, visual landmarks provided during target presentation lead to an increase in accuracy and precision of reaching movements (Krigolson and Heath, 2004; Obhi and Goodale, 2005; Krigolson et al., 2007). The effect of reduced reach endpoint variability was even more pronounced when the landmarks were placed close to the reach target (Krigolson et al., 2007). If landmarks are present while participants reach to remembered targets updated in their visual periphery, the influence of gaze-dependent spatial coding has been found to decrease suggesting a combined use of egocentric and allocentric information (Schütz et al., 2013). Such combination of egocentric and allocentric reference frames is supposed to occur after the intervening saccade at the time of action (Byrne et al., 2010) and depends on heuristics for external cue stability as well as the reliability of egocentric and allocentric cues which determines the weighting in memory-guided reaching (McGuire and Sabes, 2009; Byrne and Crawford, 2010). In addition, the proximity of the landmarks and the target seems to affect reach endpoints showing systematic distortions toward the nearest landmark (Diedrichsen et al., 2004). However, this effect only occurred when landmarks were available during target encoding but not during reaching. Moreover, structured visual background placed close to the target led to more precise reaching movements than distal visual background presumably linked to the proximity of veridical target location (Krigolson et al., 2007). The use of allocentric cues in addition to egocentric representations has even been demonstrated for imagined landmarks which were not physically present during target encoding or reaching but represented a virtual straight line (Carrozzo et al., 2002). The authors argued for the use of concurrent and independent coexisting egocentric and allocentric target representations used for memory-guided reaching.

Here we set out to address a series of controversies and gaps in the literature: (1) so far, isolated visual targets together with abstract, task-irrelevant landmarks on an otherwise blank screen have been used to investigate the underlying reference frames for reaching movements. However, it is not a given that findings from such abstract studies will hold in natural situations, where we are surrounded by a vast number of visual features creating a complex visual scene; (2) moreover, previous studies (e.g., Schenk, 2006; Zaehle et al., 2007; Thaler and Goodale, 2011a,b) explicitly asked participants to use a predefined egocentric or allocentric reference to perform the task probably covering individual spatial coding strategies. Therefore, one aim of our study was to examine the contribution of egocentric and allocentric information to reaching to images of a natural scene without biasing subjects’ behavior to use either one or the other reference frame; (3) it has been suggested that object proximity is an important factor biasing reach endpoint (Diedrichsen et al., 2004); we will challenge this view here; and (4) we will further test whether allocentric information influences reach trajectory planning (Burns and Blohm, 2010) vs. feedback-based control processes (Krigolson et al., 2007). Participants reached to a remembered location of an object on a breakfast table while we varied the location of the surrounding objects by applying a leftward or a rightward shift (allocentric cue). Spatial shifts were either applied to surrounding objects on the table which could be potential targets and were thus task-relevant (local objects) or to objects in the environment which never served as a target (global objects). Since the position of gaze, head and body were kept constant, we expected no systematic reach errors if participants relied on an egocentric target representation alone. If participants represented the target with respect to other objects on the table and/or in the environment, i.e., they used an allocentric representation, we predicted reach errors which vary as a function of object shifts. We show that memory-guided reaches to images of naturalistic environments are planned using both egocentric and local allocentric information, but not global allocentric cues.

## Materials and methods

### Participants

Data were recorded from 14 participants with normal or corrected to normal vision. One subject was excluded from further analysis because of poor fixation behavior (<1% valid trials), another subject because of frequent movement onsets while the test scene was still displayed (29.2%). The final sample consisted of 12 participants (3 female; 3 left-handed, self-report) ranging in age from 20 to 37 years (mean 24 ± 4 years). All procedures were conducted in agreement with the ethical guidelines of the local ethics committee of the University of Giessen and were approved by the Queen’s University Ethics Committee in compliance with the Declaration of Helsinki.

### Materials

**Figure 1:**
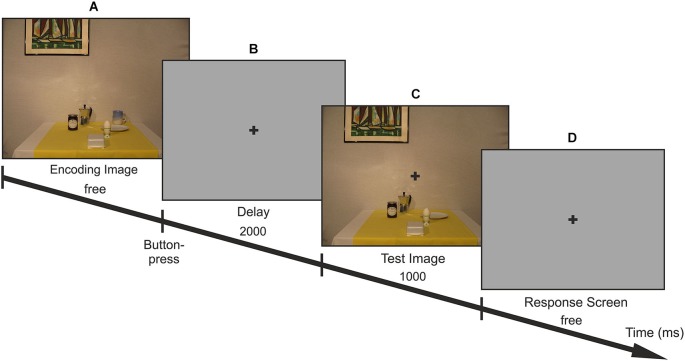
**Trial-procedure**. Participants first viewed 1 of 18 encoding images **(A)** without time limit and free gaze. After a 2 s blank gray screen **(B)** the test image appeared for 1 s **(C)** in which one of the objects on the table was missing (= target; here: cup). They were instructed to reach to the remembered target as soon as the response screen was presented **(D)**. After the encoding period, fixation had to be maintained at the fixation cross until the end of the reach.

Participants viewed photographic color images showing a breakfast scene with six *local objects* (coffee mug, plate, espresso cooker, marmalade jar, butter dish, and egg cup) on a table that was placed in front of a white wall and three *global objects* (table [T], table cloth [C], and painting on the wall [P]) in the scene (see Figure 1A). The object properties are summarized in Table 1.

**Table 1:** **Maximum height and width of objects in the scene in cm**.

**Object**	**Height**	**Width**
Plate	2.1	19
Butter dish	4.9	8.5
Marmalade jar	10.6	6.5
Coffee mug	10.3	8
Egg cup	10.1	4.1
Espresso cooker	15	15
Painting	41	51
Table	75.4	78
Table cloth	/	60

The six local objects were arranged in 18 different configurations on the table to minimize memory effects (*encoding image*). To this end, the objects were assigned to one of four possible locations in depth (8 cm, 28 cm, 48 cm, or 68 cm from the front table edge) and to a randomized horizontal position. Configurations were pseudo-randomized and fulfilled the following criteria: (i) at least one object was placed at every depth position; (ii) objects were placed with a minimum horizontal distance of 8 cm away from the edges of the table cloth in order to enable horizontal displacement on the table cloth; and (iii) <50% of each object was occluded. In addition to the encoding images, we created *test images* lacking one of the 6 local objects (= reach target). In 2/3 of the test images, local or global objects were physically displaced in the horizontal direction on the table by 8 cm either to the left or to the right (50% leftward displacement) prior to taking photographs. Due to the finite camera distance, this corresponds to different shifts on the image (and thus also on the screen), depending on the depth position of the object, i.e., whether it was located in the proximal, first medial, second medial or distal depth plane. Thus resulting visual shifts on the screen images could be 4.24°, 3.80°, 3.36° and 2.92° for proximal, first medial, second medial or distal object depth respectively. In the remaining 1/3 of the test images, the remaining objects in the scene were not shifted. In order to ensure precise and reproducible object placement in the images, a grid was projected from above on the table before the photographic image was taken with a resolution of 2048 × 1536 pixels.

In total, 342 photographic images were taken including 18 encoding images and 324 test images with 108 images without object displacement, 108 images with local object displacement and 108 images with global object displacement. Separate photographic images were taken for each target (6) in each configuration (18) and experimental condition (3; control, local and global).

### Apparatus

Stimuli were presented on a 19″ (40.64 cm × 30.48 cm) CRT monitor with a resolution of 1920 × 1200 pixels and a refresh rate of 60 Hz using the Psychtoolbox (Brainard, 1997) in Matlab (The Mathworks, Inc., Natick, MA, USA). Monitor/image edges were visible. Participants sat at a table with their head stabilized on a chin rest guaranteeing an eye-monitor distance of 47 cm. They performed the task in complete darkness but the use of a computer screen resulted in some limited illumination of the hand. Participants executed right arm reaches from an elevated start position placed 27 cm in front of the screen at the level of the lower screen edge. Reaches were recorded with an Optotrak Certus (NDI, Waterloo, ON, Canada) infrared marker-based motion tracking system with a sampling rate of 250 Hz using one marker attached to the fingertip. In order to control for correct fixation behavior, we also recorded eye movements using an EyeLink 1000 tracking system (SR Research, Osgoode, ON, Canada) with a sampling rate of 1000 Hz. Participants initiated the trials by a left-hand button press on a game controller located on the table in front of their left shoulder.

### Procedure

The trial procedure is illustrated in Figure 1. Participants started each trial by a button press with their left hand. An encoding image containing all local and global objects was displayed on the screen until participants continued the trial with a button press on the controller. They were instructed to encode the location of the local objects in the scenes while freely moving the eyes. Participants had as much time as desired and were instructed to press the game controller with the left hand in order to pursue the trial. The encoding phase was followed by a central fixation cross that appeared on a uniform gray background for 2 s prompting participants to maintain fixation at this location until the end of the reach. Then, the test image without one of the six local objects was presented for 1 s, superimposed with a fixation cross. After the test image disappeared, the fixation cross was displayed on a uniform gray background and participants were asked to reach with their right hand to the remembered location of the missing object (= target) on the screen. Thus, reaches were performed while fixating at the center of the screen and without any visual information about the scene. Whenever participants were unsure about the location of the target, they were instructed not to reach but to continue with the next trial.

**Figure 2:**
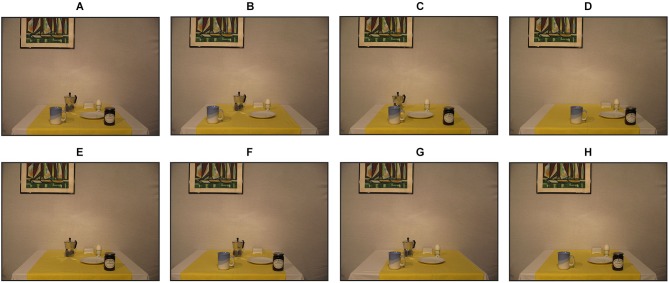
**Example images of one encoding image and seven corresponding test images. (A)** Encoding image with all six objects. **(B)** Test image of the local 1 condition (loc1) with the marmalade jar missing and the cup shifted to the left. **(C)** Local 3 condition (loc3) with missing butter dish and espresso, egg and plate shifted to the left. **(D)** Example image from the local 5 condition (loc5): The espresso cooker is missing, all other objects are shifted to the right. **(E)** Control condition with missing cup. **(F)** Global-Table (gloT) condition with the egg missing and the table shifted to the left. **(G)** Global-Table cloth (gloC) condition with the marmalade jar missing and the table cloth shifted to the right. **(H)** Global-Painting (gloP) condition with the espresso cooker missing and the painting shifted to the right.

Participants performed three experimental conditions (Figure 2). In the *allo-local* condition, we manipulated the number of local objects shifted in the scene of the test image before reaching. In particular, 1, 3 or all 5 remaining local objects were horizontally misplaced by 8 cm (in physical space) to the left or to the right (loc1, loc3, loc5) without affecting the position of the global objects. Within one trial, objects were always shifted in the same direction. In the *allo-global* condition, one global object was shifted by 8 cm (in physical space) leftwards or rightwards by leaving the location of the local objects unchanged (gloT, gloC, gloP). In the control condition, no object shifts occurred.

Each participant completed 648 trials split up in 18 blocks consisting of 36 trials each. Before the start of the experiment, each participant completed a training block of 18 control trials. Data of each subject were recorded in three 1 h sessions on different days consisting of six blocks each.

### Data reduction and statistical analyses

Data preprocessing and analyses were done using MATLAB and final inferential statistics were computed in SPSS (Version 21.0). An *α*-level of 0.05 was used for evaluating all effects.

**Figure 3:**
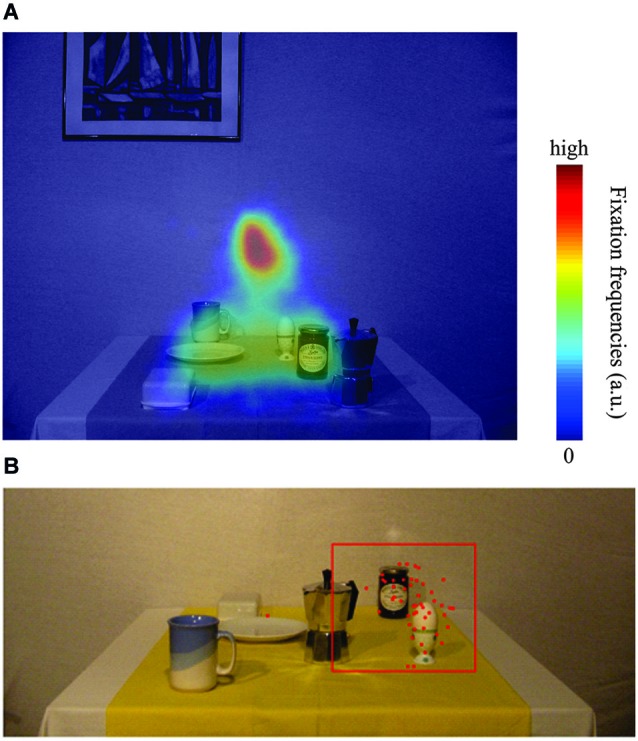
**(A)** Heatmap of relative fixation frequency during the encoding phase plotted against an example encoding image. Blue colors denote few or no fixations, whereas red colors denote many fixations in that region. **(B)** Typical example plot of reaching endpoint towards the egg in one of the 18 configurations. Red dots are reaching endpoints from individual trials (local + global). The red square represents the outlier criterion of 2.5 SD relative to the mean reach endpoint in the control condition. All data within the red square have been considered for data analysis, data points outside have been treated as outliers.

First, we analyzed eye tracking data in order to control for correct fixation. Trials were classified as invalid and excluded from further analyses if gaze deviated more than ±2.5° from the fixation location. This applied to 564 trials (7.25%). Second, reach endpoints were determined as the position where reach velocity and screen distance were minimal. Reaching endpoints in screen coordinates were then computed from camera coordinates using quaternion transformation (Leclercq et al., 2013). We excluded trials in which reach endpoints deviated more than 2.5 SD from the average reach endpoint per test image (Figure 3B). This resulted in removing 638 trials of the remaining trials (8.2%). In 187 trials (2.4%), subjects responded before the test image disappeared. To test memory-guided reaching without visual scene information, these trials were also removed for analysis. In total, 6387 out of 7776 trials remained for analysis.

#### Eye movement behavior

To investigate eye movement behavior during the scene encoding phase, we computed the relative frequency of fixations (Figure 3A). To do so, we averaged fixation positions (excluding saccades) across all encoding phase time frames and convolved the result with a Gaussian filter of 1.5° width. The result was plotted as a heat map and overlaid onto an example encoding image.

#### Allocentric weights

In order to investigate the influence of allocentric information in the scene on reach endpoints, we computed allocentric weights using linear regressions. In a first step, we calculated the group-mean reaching endpoint for every combination of object configuration and target identity in the control condition. These values served as subjective target location in the scene. In a second step, for every single reaching response in the allocentric conditions, its horizontal deviation from the subjective target location of the corresponding control image (same target and arrangement) was computed and compared to the expected allocentric deviation. Expected allocentric deviations were calculated for every test image as the average value by which the reference objects were shifted in the scene. For example, a visual leftward shift of three reference objects by 4.24°, 3.80° and 3.36° (loc3 condition; objects placed at different locations in depth) would result in an expected allocentric deviation of 3.80 cm (average of the three individual object shifts) if the target were solely represented in an allocentric reference frame, i.e., relative to other objects in the scene. In general, leftward deviations were coded as negative values and rightward deviations as positive values. In a third step, the observed horizontal deviation from the subjective target location for a leftward and for a rightward shift of the same target in the same arrangement were plotted against the expected allocentric deviations for each individual and each allocentric condition. Finally, a regression line was fitted to the data and the slope of the regression line determined the allocentric weight.

We applied one-sampled *t*-tests to examine whether individual local and global allocentric weights significantly differed from 0. Since allocentric weights are computed on the basis of the results of the control condition, a test against zero corresponds to a statistical comparison to the control condition. To compare individual allocentric weights across conditions, we then computed one-way repeated measures ANOVAs with three levels for the local condition (loc1, loc3, loc5) and the global condition (gloT, gloC, gloP), separately. Significant results were followed-up with *post-hoc* *t*-tests. Based on our hypotheses, *t*-tests were calculated one-sided and corrected for multiple comparisons using Bonferroni-Holm correction.

#### Response latency and movement time

To test for differences in movement initiation and duration depending on the experimental conditions, we examined response latencies and movement times respectively. Response latencies were determined as the time from the disappearance of the test image until the start of the reaching movement which was defined as the point in time when the right index finger exceeded a velocity of 50 mm/s for 200 ms. Movement time was determined as the time from the start of the movement until its end defined as the time point when the velocity of the index finger fell below 50 mm/s for 100 ms and distance to the screen was minimal. Individual median response latencies and movement times were compared between the experimental conditions by computing separate one-way repeated measures ANOVAs with four levels for the local condition (loc1, loc3, loc5, control) and for the global condition (gloT, gloC, gloP, control). Two-sided *post-hoc* *t*-tests were calculated and corrected for multiple comparisons using Bonferroni-Holm correction.

#### Frequency of no-reach responses

We instructed participants to perform no reach movement if they were uncertain about the location and/or identity of the target. Frequency of trials in which subjects did not respond was computed per condition and tested against the assumption that those trials are equally distributed across all conditions by using a Friedman’s test.

#### Reach trajectories

To determine whether allocentric influences were part of the overall movement plan or whether they emerged only during online corrections (cf. Krigolson et al., 2007; Burns and Blohm, 2010), we analyzed reaching trajectories using functional data analysis (FDA; Ramsay and Silverman, 2005). Some trials were excluded from the analysis due to the following reasons: (a) less than 50 data frames were collected per reaching movement due to Optotrak marker visibility problems; (b) moving velocities exceeded 600 cm/s during one reaching movement; and (c) trials lacked more than 20 consecutive data frames. Following these criteria only three trials (<0.1%) were discarded.

First, we shifted the movement onset (i.e., the first data frame) of each trajectory to the coordinate point 0/0/0 (*x*-, *y-*, *z*-direction in 3D Cartesian space) and aligned the subsequent data frames. Second, we spatially normalized the trajectories by fitting order 6 splines to each of the three dimensions (*x,y,z*) with a spline at every data frame. Third, we smoothed the data using a roughness penalty on the fourth derivative and λ = 1^-10^ (within 0.008 of the generalized cross-validation estimate). Out of this mathematical definition we evaluated for each trajectory 1200 equally spaced data points. Then, 120 out of 1200 points were extracted resulting in spatially normalized trajectories. This procedure had also the advantage that missing data frames within one reaching movement were interpolated (for further details see also, Chapman and Goodale, 2010). As reaching endpoints differed between different stimulus’ images (due to different target locations on the screen) within one condition, trajectories had to be rotated to one single reaching endpoint per condition to be able to average reach trajectories. Therefore each trajectory was transformed to the polar coordinate system. For every possible combination of object arrangements and targets, we calculated the mean angle of the last data point of the control conditions for every subject. This value was then subtracted from every angle value of the control condition and any other condition of the corresponding arrangement-target combination, resulting in a rotation of the trajectories of the control condition to the center of the display and a respective rotation of the trajectories from the other conditions. Consequently, the distances and proportions between control trajectories and the trajectories from other conditions remained unaffected. Afterwards the rotated trajectories were converted back to the Cartesian coordinate system. Finally we averaged trajectories over every condition for every subject.

For statistical analysis the preprocessed, normalized and averaged trajectories were entered into four functional-ANOVAs (Ramsay and Silverman, 2005), two for global and two for local conditions including one for right- and one for leftward object shifts. The functional-ANOVA models were single factor designs with four levels (control, loc1, loc2, loc3 and control, gloT, gloC, gloP). Functional pairwise comparisons (equivalence to a paired *t*-test) between the control condition (no object shift) and every experimental condition (with object shift) were conducted *post-hoc* (one comparison for each shift direction).

## Results

### Eye movement behavior

In the present study we investigated whether or not allocentric coding schemes are used when people reach to remembered targets in a natural scene. We manipulated the location of the reference objects by shifting the objects to the left or to the right before reaching. Reference objects were either potential reach targets (local condition) or other objects in the scenes (global condition). First, we sought to quantify eye movement behavior during the encoding phase. Figure 3A illustrates the relative frequency of fixations overlaid on an example encoding image (see Section Materials and Methods for details). Clearly, participants visually explored relevant portions of the image, i.e., local object regions where potential reach targets were located. The screen center, the position of the future fixation cross, naturally resulted in the most frequent fixation location (red). Figure 3B depicts a typical example of individual reach endpoints for one participant and the applied exclusion criteria towards one target (egg). Clearly, only real outliers were removed.

### Allocentric weights

**Figure 4:**
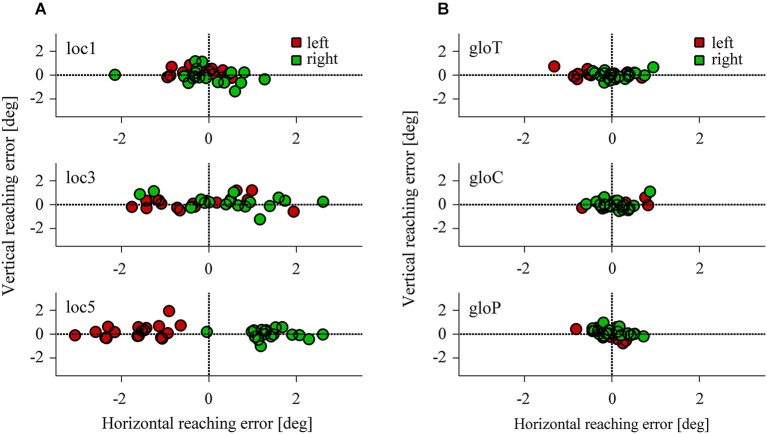
**Horizontal and vertical reaching error in the local (A) and the global conditions (B)**. Each data point represents the average reaching endpoint for one test image across participants. Red colors indicate a leftward, green colors a rightward target shift.

Figure 4 represents the reach endpoints for all participants observed in the local and global conditions. As the overall pattern shows, reach endpoints were influenced by left- and rightward shifts of three or five reference objects in the local conditions (Figure 4A) but were hardly affected in the local and global conditions when only one reference object was shifted (Figure 4B). In particular, reach errors were distributed along the horizontal axis and increased with the number of local objects shifted in the scene.

**Figure 5:**
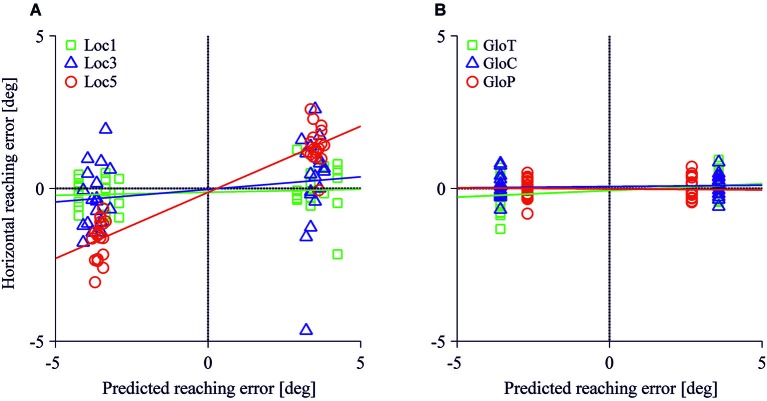
**Horizontal reaching errors as a function of predicted allocentric reaching errors for the local (A) and the global conditions (B)**. Each symbol specifies mean reach endpoints for one test image. Colored lines represent regression fits for each allocentric condition.

Figure 5 displays the observed horizontal reach errors as a function of the predicted allocentric reach errors for each test image. Reach errors varied within the expected direction of the shift of the reference objects in the loc5 and loc3 conditions where 5 or 3 local objects were shifted before the reach. The allocentric weights ranged between 1% to 43% in the local conditions and 1% to 4% in the global conditions. Table 2 summarizes the mean (SD) reach errors for each individual participant and for loc3 and loc5 conditions separately. A leftward shift of the reference objects resulted in reach endpoints left of the target location and vice versa. This was confirmed by the allocentric weights (= slope of the regression line) which significantly differed from 0 in the loc5 (*t*_(11)_ = 9.90, *p* < 0.001) and the loc3 (*t*_(11)_ = 2.43, *p* = 0.017) conditions. We found a smaller but non-significant effect for the gloT condition where the table was shifted in the scene (*t*_(11)_ = 2.36, *p* = 0.019; critical *p*-value = 0.0166).

**Table 2:** **Mean (SD) reaching endpoints relative to control condition for every participant in the loc3 and loc5 condition, split up by the direction of the allocentric shift**.

	**Loc3**		**Loc5**	
**Subject**	**Left**	**Right**	**Left**	**Right**
1	1.16 (4.65)	0.11 (4.91)	−1.78 (4.14)	0.22 (4.56)
2	−1.73 (3.03)	−0.41 (3.38)	−3.09 (1.95)	0.92 (2.46)
3	−1.12 (2.64)	−0.37 (2.19)	−2.89 (1.51)	1.21 (1.37)
4	−1.16 (3.21)	−1.29 (2.78)	−1.94 (1.25)	0.60 (1.55)
5	−2.39 (1.54)	0.29 (2.00)	−2.22 (2.67)	1.32 (1.40)
6	0.09 (2.79)	−0.18 (1.66)	−1.44 (1.11)	0.66 (1.50)
7	−0.75 (1.31)	0.82 (2.04)	−1.27 (0.99)	2.82 (1.17)
8	−0.45 (2.44)	−0.04 (1.22)	−1.09 (1.13)	0.39 (1.69)
9	0.45 (1.38)	0.76 (2.91)	0.02 (1.01)	2.06 (1.47)
10	0.17 (1.52)	1.86 (1.57)	−1.18 (1.10)	2.83 (0.98)
11	−0.59 (1.66)	0.56 (1.67)	−0.60 (0.93)	1.58 (1.33)
12	0.31 (2.41)	0.13 (3.15)	−0.72 (1.80)	0.60 (2.90)

*Each data point is based on 36 trials (minus disregarded trials). Negative values indicate a leftward shift relative to control condition. All values are reported in degree visual angle*.

**Figure 6:**
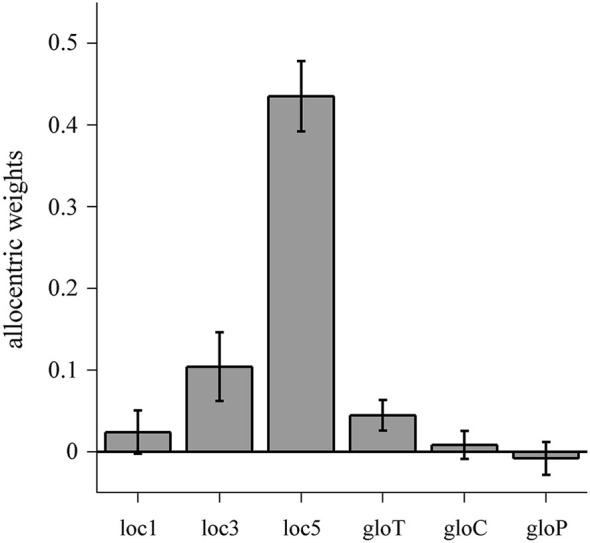
**Allocentric weights for the allo-local and allo-global conditions**. Data are averaged over individual allocentric weights with error bars denoting one standard error of variability between observers. Individual allocentric weights range from −0.15 to 0.18 (loc1), −0.14 to 0.39 (loc3), 0.21 to 0.61 (loc5), −0.06 to 0.14 (gloT), −0.09 to 0.10 (gloC) and −0.16 to 0.08 (gloP).

To compare the individual allocentric weights within the allo-local and the allo-global conditions, we computed one-way repeated measures ANOVAs which revealed a significant main effect of condition for allo-local (*F*_(2,22)_ = 59.35, *p* < 0.001) but no effect for allo-global (*F*_(2,22)_ = 2.438, *p* = 0.111). *Post-hoc* *t*-tests indicated that allocentric weights in the loc5 condition were significantly higher than in the loc3 (*t*_(11)_ = 8.935, *p* < 0.001) and the loc1 (*t*_(11)_ = 9.448, *p* < 0.001) conditions. In addition, allocentric weights in the loc3 condition were higher than in the loc1 condition (*t*_(11)_ = 2.348, *p* = 0.019). Thus, allocentric weights increase with an increasing number of local reference objects shifted in the horizontal plane (Figure 6).

It has previously been shown that landmarks can influence reach trajectories and that this effect is distance dependent (Diedrichsen et al., 2004). Therefore, we also tested for the effect of proximity in the loc1 and loc3 conditions by correlating the observed reaching error with the mean distance of the shifted object/s with respect to the target. However, we could neither find a correlation for the loc1 (*r* = −0.09, *p* = 0.615) nor for the loc3 (*r* = −0.01, *p* = 0.962) conditions.

### Response latency and movement time

**Figure 7:**
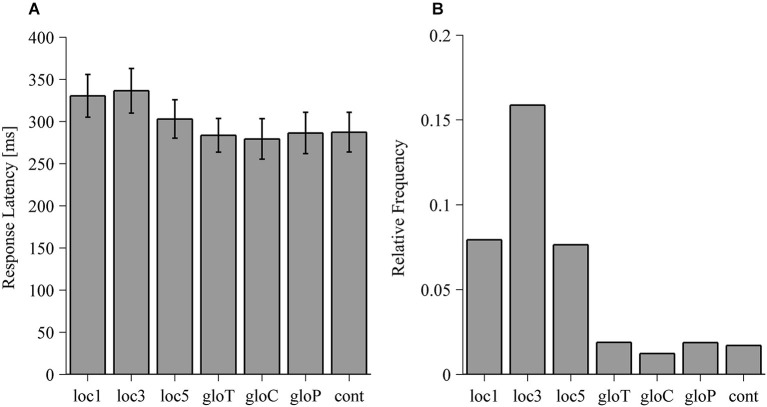
**(A)** Response latencies of reaching movements in ms for the local, global and control (cont) conditions. Values are averaged across median response latencies of individual observers. Error bars denote one standard error of variability between observers. **(B)** Relative frequency of trials where participants with no reach response for the local, global and control (cont) conditions. For each condition, the relative frequency is computed as the amount of trials without a reaching response divided by the total amount of trials in that condition.

Response latencies of reaches for the allo-local and the allo-global conditions are illustrated in Figure 7A. Response latencies did not significantly differ between the allo-global conditions (*F*_(3,33)_ = 0.372, *p* = 0.774) but significantly differed between the allo-local conditions (*F*_(3,33)_ = 14.54, *p* < 0.001). In comparison to the control condition, reaches were slower in the loc1 (*t*_(11)_ = 5.643, *p* < 0.001) and the loc3 (*t*_(11)_ = 6.64, *p* < 0.001) conditions. Moreover, reaches in the loc3 condition were also initiated more slowly than in the loc5 condition (*t*_(11)_ = 3.616, *p* = 0.004). Movement times did neither vary between allo-local conditions (*F*_(3,33)_ = 0.560, *p* = 0.645) nor between allo-global conditions (*F*_(3,33)_ = 0.44, *p* = 0.726).

### Frequency of no-reach responses

To assess task difficulty, we tested whether the frequency of trials in which subjects did not respond differed across all conditions and thus violates the assumption of equal trial distribution across conditions. The results of the Friedman test rejected the assumption that those trials are equally distributed across all conditions (*χ*^2^ = 46.6, *p* < 0.001). As depicted in Figure 7B, participants showed more frequent no reaching responses in the local compared to the global conditions with the highest frequency in the condition where three local objects were shifted (loc3).

### Reach trajectories

**Figure 8:**
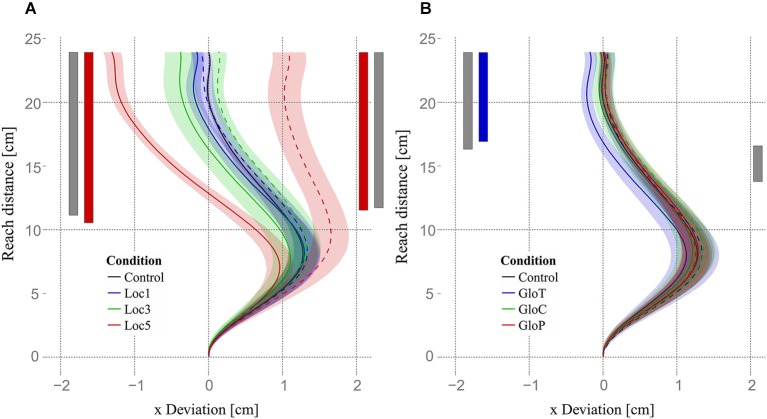
**Trajectories and results of functional analysis**. Mean trajectories of all subjects are plotted as the deviation on the *x*-axis (parallel to the screen) against the reaching distance (axis orthogonal to the screen). Trajectories for leftward object shifts are plotted with solid lines and rightward object shifts with dashed lines. Gray bars indicate the area where trajectories of left- or rightward shifts for local or global conditions showed a significant main effect. **(A)** Mean trajectories for all local conditions and the control condition are displayed. Red bars indicate the area where leftward and rightward shifts of the loc5 condition significantly differed from the control condition. **(B)** Mean trajectories for all global conditions and the control condition. The blue bar indicates the area where leftward shifts in the gloT condition significantly differed from the control condition. Shaded areas in **(A)** and **(B)** express one standard error of the mean of the corresponding mean trajectory.

To examine whether reaching errors due to allocentric object shifts emerged early during the reaching movement (due to different motor plans) or late during the reaching movement (due to error correction mechanisms), we used four functional ANOVAs (one for each experimental condition and shift direction) and functional pairwise comparisons to compare reaching trajectories of different allocentric conditions and the control condition. The functional ANOVAs revealed that trajectories of local object shifts differed in the horizontal plane (*x*-axis, parallel to the screen). Trajectories for both leftward and rightward shifts started to differ roughly at half-distance (≈48.75% = 11.7 cm) of the reach trajectory (Figure 8A, significant regions indicated by the gray vertical bars). Functional ANOVAs for global object shifts showed significant differences for leftward shifts starting from roughly the last third (68.3% = 16.4 cm) up to the end and for rightward shifts just for a small area right after half-distance (from 57.5% = 13.8 up to 68.3% = 16.4) of the reaching movement. Subsequent functional pairwise comparisons between every local condition and the control condition for the two shift directions showed that only trajectories in the loc5 condition differed significantly from the control condition. Loc5 trajectories for leftward and for rightward shifts started to differ slightly earlier than half-distance of the reaching movement (leftward: 43.3% = 10.4 cm; rightward: 48.3% = 11.6 cm). Differences increased until the end of the movement (Figure 8A, indicated by the red vertical significance bars). Functional pairwise comparisons for global conditions revealed only a significant difference between gloT and the control condition for leftward object shifts for roughly the last third of the reaching movement (starting from 70.8% = 17 cm till the end; Figure 8B, indicated by the blue vertical significance bar).

## Discussion

In this study, we investigated the use of egocentric and allocentric information during memory-guided goal-directed reaching using a naturalistic visual scene. Allocentric information was varied by shifting objects on the table (local objects) or objects in the environment (global objects) leftwards or rightwards after scene encoding and before reaching. Memory-guided reaching movements were performed without visual information about the scene while gaze and body position remained fixed. We predicted accurate reaching movements if participants relied only on egocentric object coding, i.e., representing the target relative to gaze or body position, and systematic shifts of reach endpoints if they used allocentric cues (local or global) for goal-directed reaching. Our results demonstrated that reach endpoints varied as a function of objects shifted in the scene. The more local objects were horizontally misplaced the larger were the reach errors in the direction of the objects shifted. The present findings suggest that allocentric cues are indeed used during goal-directed reaching, but only if a substantial change of allocentric information is present in complex visual scenes.

Previous studies consistently reported that reach targets are represented relative to gaze direction, i.e., in an egocentric frame of reference (e.g., Henriques et al., 1998; Medendorp and Crawford, 2002). Beyond egocentric coding, allocentric cues also contribute to reaching movements as has been demonstrated in studies using visual landmarks (Obhi and Goodale, 2005; Byrne and Crawford, 2010; Byrne et al., 2010), imagined landmarks (Carrozzo et al., 2002) or structured visual backgrounds (Krigolson and Heath, 2004; Krigolson et al., 2007). While these studies examined reaching movements in rather unnatural tasks using isolated visual targets presented together with abstract, task-irrelevant landmarks, here we studied reaching behavior with more naturalistic stimuli by using photographic images of a breakfast scene. Despite the stable and reliable egocentric information of body and gaze position, we found large effects of allocentric cues on reach endpoints in line with the previous findings based on less ecologically valid experimental tasks (e.g., Byrne and Crawford, 2010). Since the target was defined as the missing local object in the shifted target scene, object shifts seem to be incorporated into the memory representation of the target established during scene encoding resulting in a combined representation which is used for calculating the reach plan. This is supported by the reaching trajectories in the object shift condition (loc5) which started to deviate from the no-shift condition early after reach onset. In sum, our results suggest that allocentric cues are even effective if they are provided after target encoding.

The present results demonstrated that the number of local objects shifted in the scene systematically affected reaching movements. We found larger distortions of reach endpoints with an increasing number of local objects shifted in the scene. Reach errors were most pronounced when all remaining local objects (loc5) were shifted, intermediate when three local objects (loc3) were shifted and absent for shifts of one local object (loc1). This result implies that substantial changes of allocentric cues in complex visual scenes are required to influence reaching movements. It is important to note that after object shifts the spatial relations between the objects in the loc5 condition remained constant while they completely changed in the loc3 condition. This resulted in a higher number of no-response trials and slower response latencies in the loc3 condition indicating higher task difficulty. Nevertheless, allocentric coding was still present in the loc3 condition, but the effect was diminished compared to the loc5 condition. Based on the present data, we cannot disentangle whether the reduced effect of allocentric coding is caused by larger task difficulty or fewer changes in the scene image. Previous findings on the Roelofs effect argue for the latter factor showing that the amount of a perceived target displacement when the whole frame around the target was shifted equaled the sum of a perceived target shift when only parts of the frame were shifted (Walter and Dassonville, 2006). Accordingly, we observed that allocentric weights were highest in conditions, when five local objects were shifted and lowest, when only one object was moved with the weights of three shifted local objects in between. We exclude a potential effect of proximity of target and allocentric cues on reach endpoints (c.f., Diedrichsen et al., 2004) because local object shifts appeared in the immediate vicinity of the target. Thus, we suggest that in a realistic visual environment it is the number of changed allocentric cues rather than distance that determines integration weight.

Local objects might also function as potential obstacles in real world situations which are especially important for movement programming. Obstacles constitute spatial constraints on movement execution and thus are not considered as distractors but rather as task-relevant non-target information (Tresilian, 1998) which is represented together with the target information in the attention system (Tipper et al., 1997; Baldauf and Deubel, 2010). As a consequence, the presence of obstacles requires additional anticipatory processing of movements leading to slower movement initiation (Saling et al., 1998; Biegstraaten et al., 2003). Accordingly, we observed longer response latencies when local objects (loc1 and loc3) were shifted, but not for global object shifts.

The absence of an influence of global allocentric cues on reach endpoints can be explained by multiple factors. First, the changes of global objects in the scene were undersized due to only one global object being shifted (instead of multiple as in the local conditions). Therefore, global conditions might be more similar to the loc1 condition. One can speculate that an increase in the number of shifted global objects might lead to similar results as we observed for the local object shifts. Second, it is also possible that it is the object displacement relative to object size that plays a role, in which case smaller objects should have larger influences on allocentric coding. Third, we cannot entirely rule out that the visibility of the frame of the presentation screen throughout the experiment has acted as a strong global allocentric cue. Since the screen never moved but the frame of the screen was a very salient visual feature (i.e., high contrast), it might have overridden more subtle global allocentric cues within the images. Fourth, local and global objects differed in task relevance, in the way that local objects represented potential reach targets in contrast to global objects which never served this function. This information was given by task instruction and thus may have influenced strategic behavior. Task relevance has been shown to affect overt attention in naturalistic tasks resulting in more fixations on task-relevant than task-irrelevant objects (Land and Hayhoe, 2001; Ballard and Hayhoe, 2009). These findings are consistent with the fixation behavior we observed during the encoding phase which was spatially restricted to locations of the local objects. Fixations also frequently occurred at the table/table cloth placed right underneath the local objects; however, these global objects did not affect reaching behavior. In support of this finding, previous work demonstrated that object features which are task-irrelevant are not attended even if the respective object is fixated (Triesch et al., 2003). Together with the fact that working memory capacity for spatial information is limited to up to 4 items (Luck and Vogel, 1997) and retention of task-relevant objects is prioritized (Maxcey-Richard and Hollingworth, 2013), it is conceivable that participants encoded the location of local objects, i.e., task-relevant information, which were then incorporated into the reach plan while ignoring the location of the global objects, i.e., task-irrelevant information in the environment. Whether or not task relevance of allocentric information is a central factor in reach planning should be examined in future studies. Finally, the global allocentric cues lacked of a causal relationship to the reach target as discussed in the next paragraph.

We believe that our findings can be explained in the framework of causal Bayesian integration (Körding and Tenenbaum, 2007; Körding et al., 2007). The gradual increase of allocentric cue effects with the number of shifted local objects is consistent with more reliable allocentric cue information when more local objects are shifted. In that sense, the more local objects are shifted, the smaller the variance associated with allocentric information and thus the higher the allocentric weight in the integration of egocentric (probably body and gaze) and allocentric position. But how does this explain the absence of global allocentric cue effects? We believe that the concept of causality in Bayesian integration might be a key in understanding this. First, one can argue that there is no real causal link between the global objects and the local objects, as the picture frame is totally task-irrelevant and the exact position of the table and table cloth are not important, unless local objects had been positioned at the edge (and could thus fall off), which was not the case. Second, the spatial extend of the table and table cloth might have simply resulted in less precise positional information due to their large spatial extent. Third, and maybe more importantly, when the table cloth or table moved, local objects stayed fixed in space (i.e., did not move with the table and table cloth). Thus, the causal link between table/cloth and local objects on the table was broken, since normally objects would move with the table/cloth. In that case, causal Bayesian integration discounts any global allocentric cue effects due to a lack of a causal relationship between table/cloth movement and target location.

Our observations that movement endpoints are systematically shifted by local allocentric cues could result from two different sources: reach trajectory planning (Burns and Blohm, 2010) or feedback-based control processes (Krigolson et al., 2007). Indeed, allocentric information could be included in the reach plan right from the start as is the case in visual-proprioceptive integration (Sober and Sabes, 2003, 2005; Burns and Blohm, 2010), in which case one would expect manifestations of allocentric influences on the reach plan early on in the reach trajectory. Alternatively, allocentric information could only be incorporated during feedback corrective processes (i.e., later on in the movement), which would be consistent with observations of allocentric visual background influences on reaches (Krigolson et al., 2007). Our data on reaching trajectories is consistent with the former hypothesis and shows that local allocentric information might influence reach planning differently than allocentric background information.

In the present study we examined egocentric-allocentric cue integration for memory-guided (not visually-guided) reaches. Reaches were performed immediately after the presentation of the test scene; a condition which is usually defined as immediate reaching (cf. Bridgeman et al., 2000; Hay and Redon, 2006). However, here we asked participants to reach to the missing object in the test scene which required to build up representations of potential reach targets during the encoding scene which were then updated on the basis of the test scene after a 2 s delay. Delay is believed to have an important influence on spatial coding. For example, Hay and Redon (2006) found that delayed reaching accuracy declined in darkness but remained constant when a structured visual background was available. They explain their findings with a decaying egocentric representation and a more permanent allocentric representation of target location. This is also consistent with observations that visual landmarks increase space constancy (Deubel et al., 2010) and decrease egocentric, gaze-dependent coding of reach targets (Schütz et al., 2013). Furthermore, allocentric information has a stronger impact on delayed than immediate reaches showing increased reach errors in the direction of a shifted landmark with longer delays between stimulus offset and motor response (Bridgeman et al., 1997, 2000). An interesting prediction from these findings is that shorter (resp. longer) delays should lead to lower (resp. higher) allocentric weights because egocentric information is initially more accurate but decays faster than allocentric information.

Overall, we have shown that allocentric information is used by the brain to plan memory-guided reaches towards targets in naturalistic visual images. Our data is generally consistent with Bayesian causality principles and demonstrates that egocentric-allocentric cue integration is highly flexible and task-dependent. It would be interesting to further examine the role of causality in egocentric-allocentric cue integration, in particular with respect to the causal relationship between visual landmarks.

## Conflict of interest statement

The authors declare that the research was conducted in the absence of any commercial or financial relationships that could be construed as a potential conflict of interest.
